# Supplementary material for: Genes linked to schistosome resistance identified in a genome-wide association study of African snail vectors
Source: Nat Commun. 2025 Jul 27;16:6918. doi: 10.1038/s41467-025-61760-8 (PMC12297450; doi:10.1038/s41467-025-61760-8)
Supplement: Supplementary file 1 — Supplementary Information [file 41467_2025_61760_MOESM1_ESM.pdf]

Supporting Information for:

Genes linked to schistosome resistance identified in a genome-wide association study of African snail vectors

Tom Pennance[a,#], Jacob A Tennessen[b,#], Johannie M Spaan[a], Tammie J. McQuistan[a], George Ogara[c], Fredrick Rawago[c], Kennedy Andiego[c], Boaz Mulonga[c], Meredith Odhiambo[c], Martin W Mutuku[d], Gerald M Mkoji[d], Eric S Loker[e], Maurice R Odiere[c], and Michelle L Steinauer[a,*]

[a] College of Osteopathic Medicine of the Pacific – Northwest, Western University of Health Sciences, Lebanon, OR, USA
[b] Harvard T.H. Chan School of Public Health, Boston, MA, USA
[c] Centre for Global Health Research, Kenya Medical Research Institute (KEMRI), P. O. Box 1578-40100, Kisumu, Kenya
[d] Centre for Biotechnology Research and Development, Kenya Medical Research Institute (KEMRI), P.O. Box 54840–00200, Nairobi, Kenya
[e] Department of Biology, Center for Evolutionary and Theoretical Immunology, Parasite Division Museum of Southwestern Biology, University of New Mexico, Albuquerque, New Mexico, USA
[#] Authors contributed equally.
* To whom correspondence may be addressed
Email: msteinauer@westernu.edu

This PDF file includes:

Figures S1 to S20
SI References

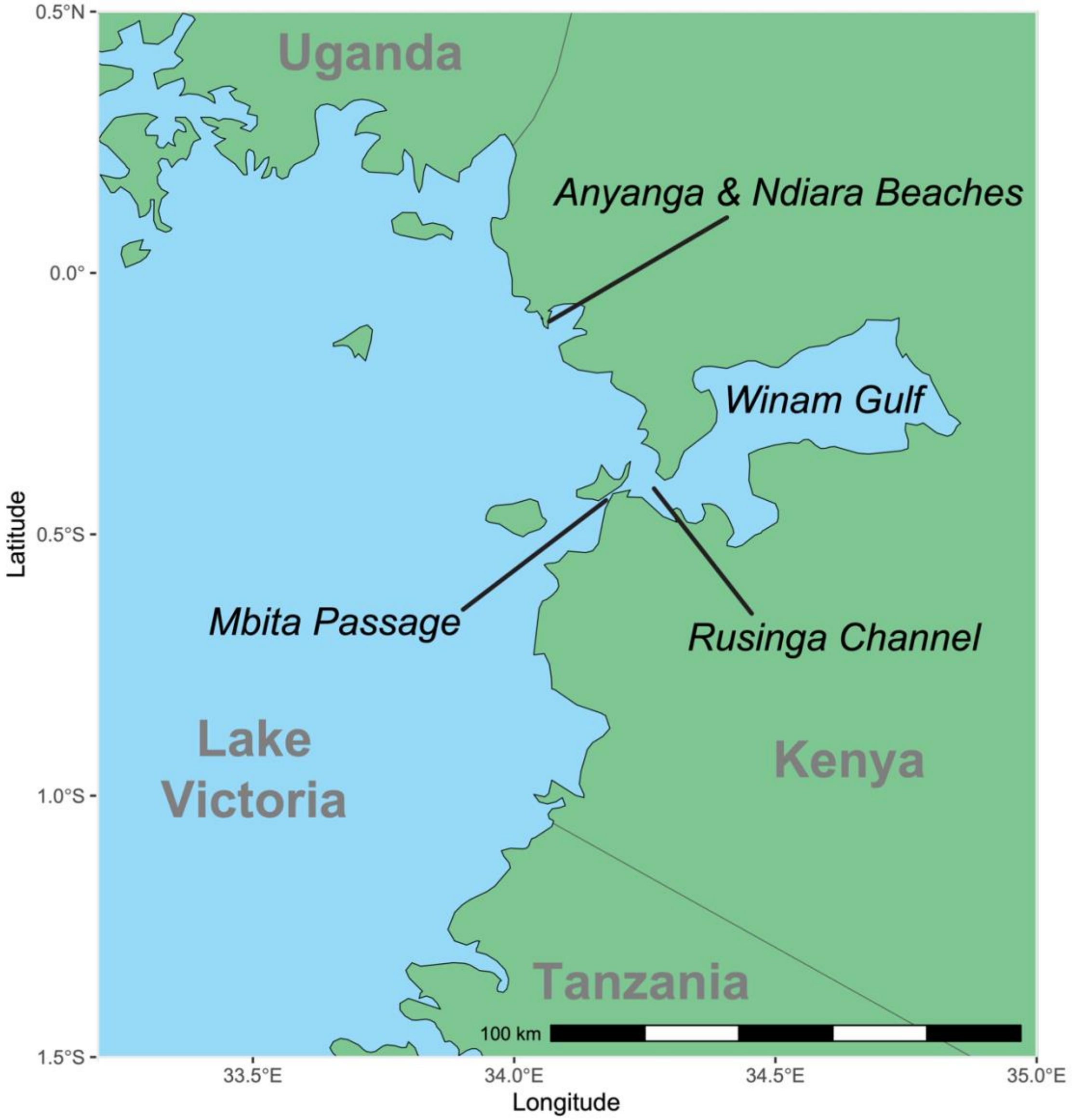

Fig. S1. Map of northeastern Lake Victoria, showing our collection sites (Anyanga and Ndiara Beaches, Kanyibok, Kenya) and nearby geographical features that may influence local population structure (Winam Gulf, Mbita Passage, and Rusinga Channel).

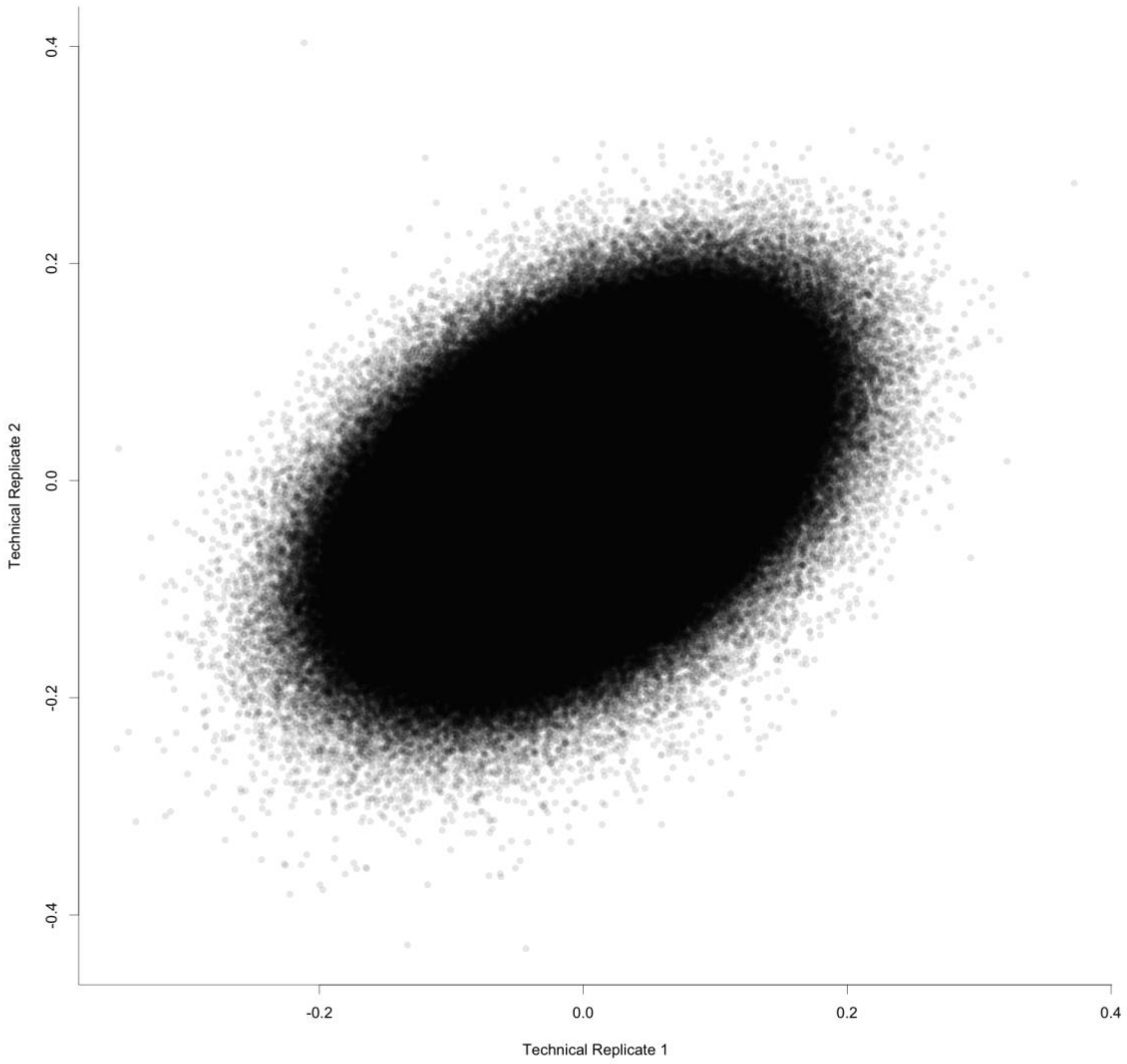

Fig. S2. Plot of differences in allele frequency between positive and negative pools, across technical replicates. The x-axis shows the difference in frequency between true positives and true negative at all variants for technical replicate 1. The y-axis shows the equivalent values for the same variants in technical replicate 2. Values between technical replicates show a significant positive correlation (Simple linear regression, $R^2 = 0.22$, $p < 1e\text{-}300$).

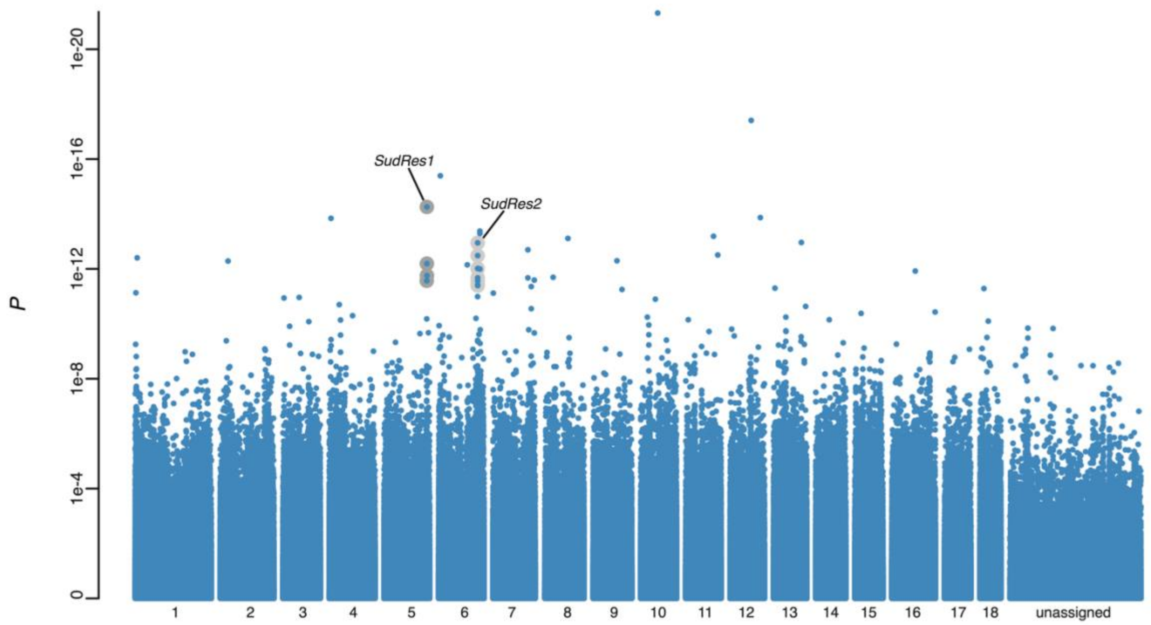

Fig. S3. Re-analysis of pooled-GWAS after controlling for depth heterogeneity. All sites were subsampled to 300 true negative reads and 300 true positive reads; sites failing to meet this depth were discarded. While absolute P values are lower than Fig. 1 due to reduced sample size, the relative positions of outliers are largely the same. Critically, outlier variants still occur at both *SudRes1* (circled in dark grey) and *SudRes2* (circled in light grey) indicating that these loci are not artifacts of read depth.

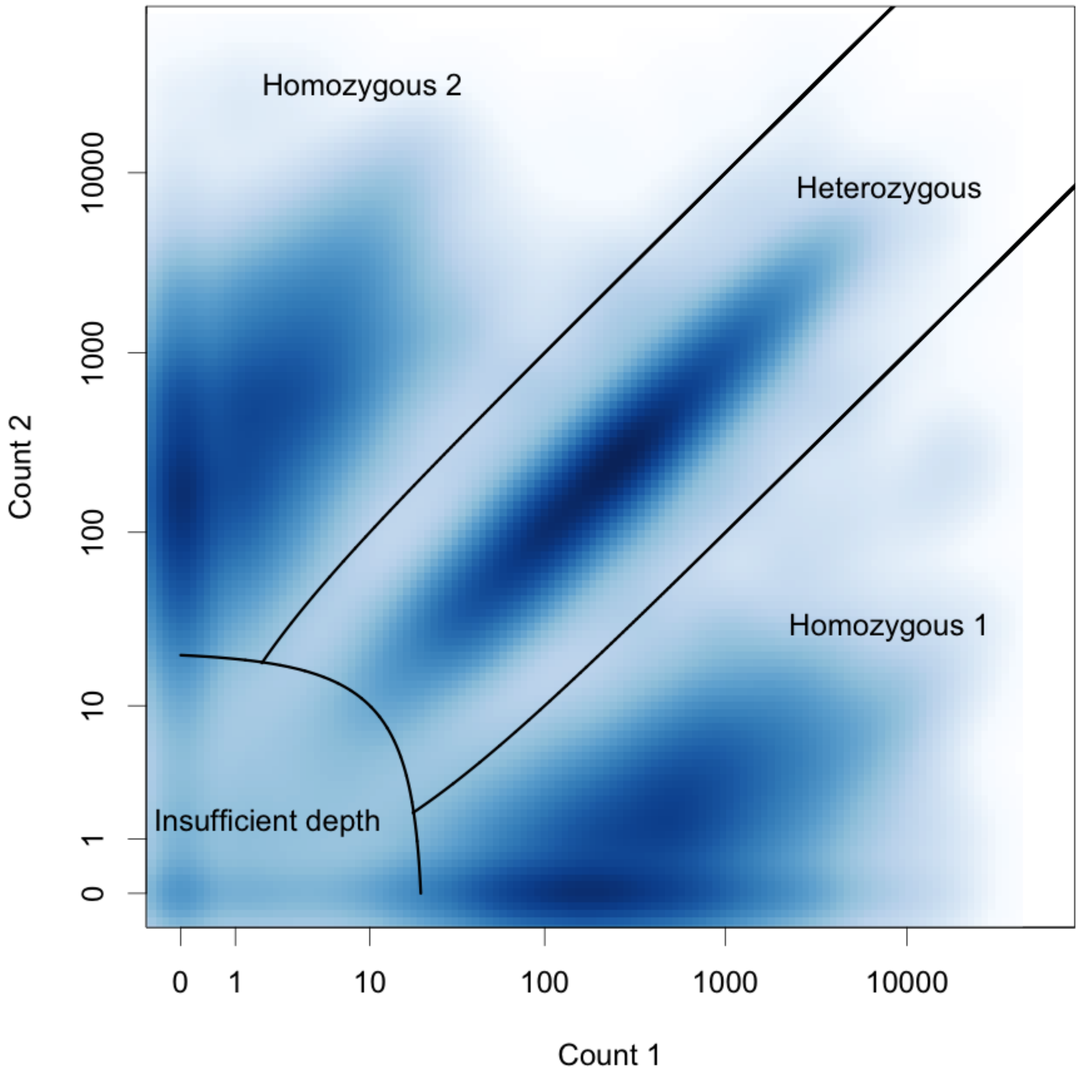

Fig. S4. Density plot of allele counts at amplicon genotypes. Alleles at each marker are arbitrarily designated 1 or 2. The black lines show our genotyping thresholds. Genotypes with fewer than 20 total reads are counted as missing (insufficient depth). If the ratio between the less common allele and the more common allele is >10% that genotype is called as heterozygotes, otherwise it is called as homozygous for the more common allele. This approach successfully distinguishes the three main clusters corresponding to the three possible genotypes.

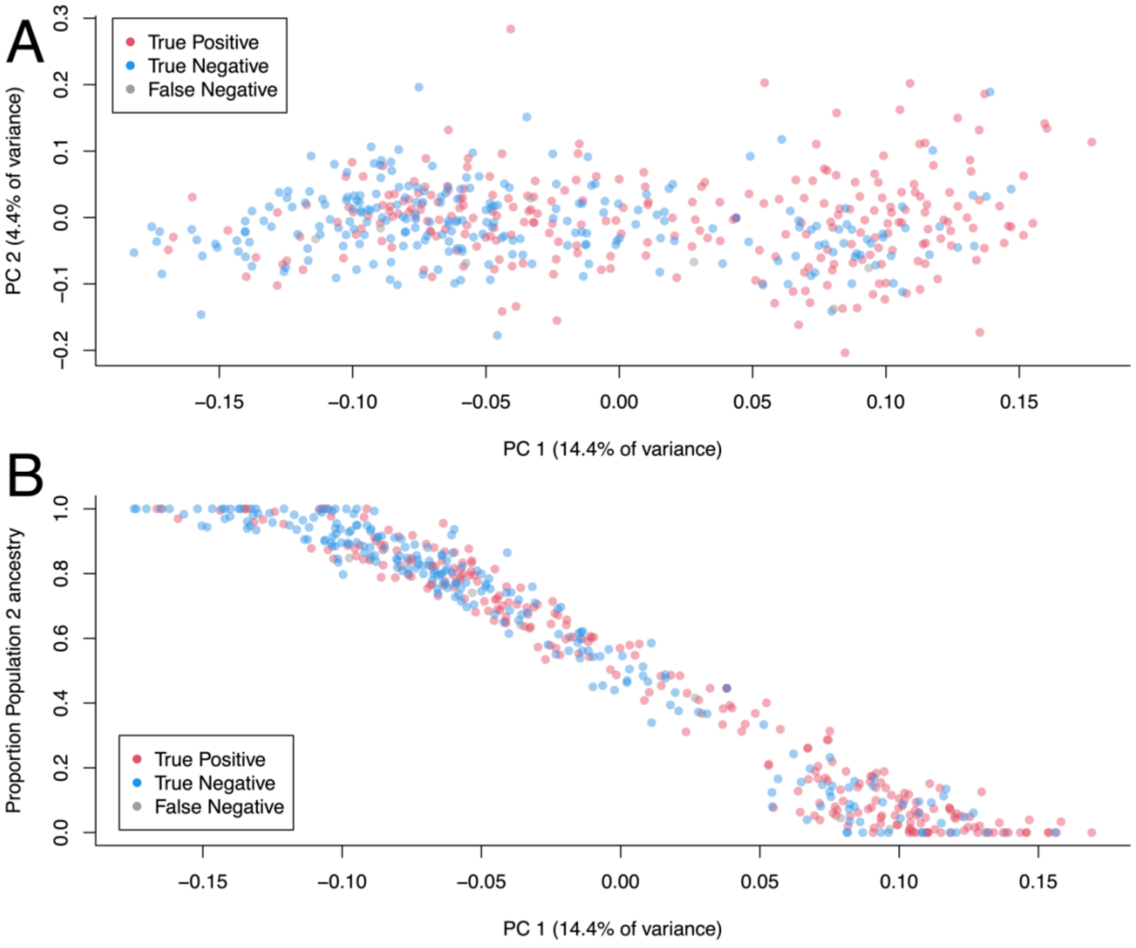

Fig. S5. Principal Component Analysis of amplicon genotypes recapitulates ancestry inference from ADMIXURE (15). We included all 201 ‘neutral’ (non-candidate) loci and all n=503 *B. sudanica* individuals (missing data imputed as heterozygous). Samples are colored based on phenotype. (**A**) PCA of first two principal components using PC-AiR (17). (**B**) Principal component 1 plotted against ancestry as estimated from ADMIXTURE.

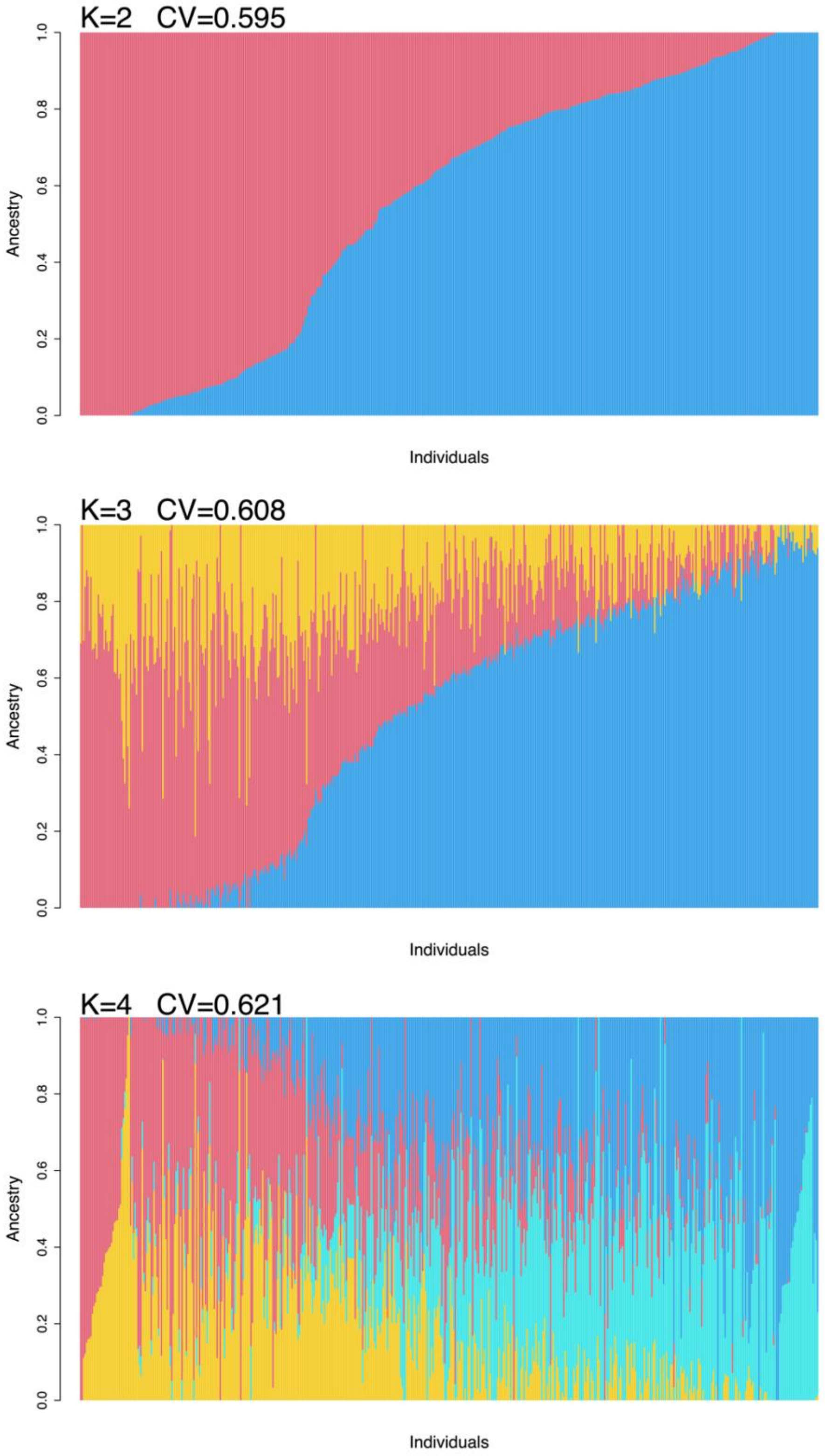

Fig. S6. ADMIXTURE (15) analysis with K of 2 (top, equivalent to Fig. 2A), K of 3 (middle), or K of 4 (bottom), n=503. We first calculated ancestry for unrelated individuals identified by PC-AiR (17), then projected ancestry for the remaining samples. Cross-validation (CV) error was lowest for K of 2, justifying our use of this model in validation analysis.

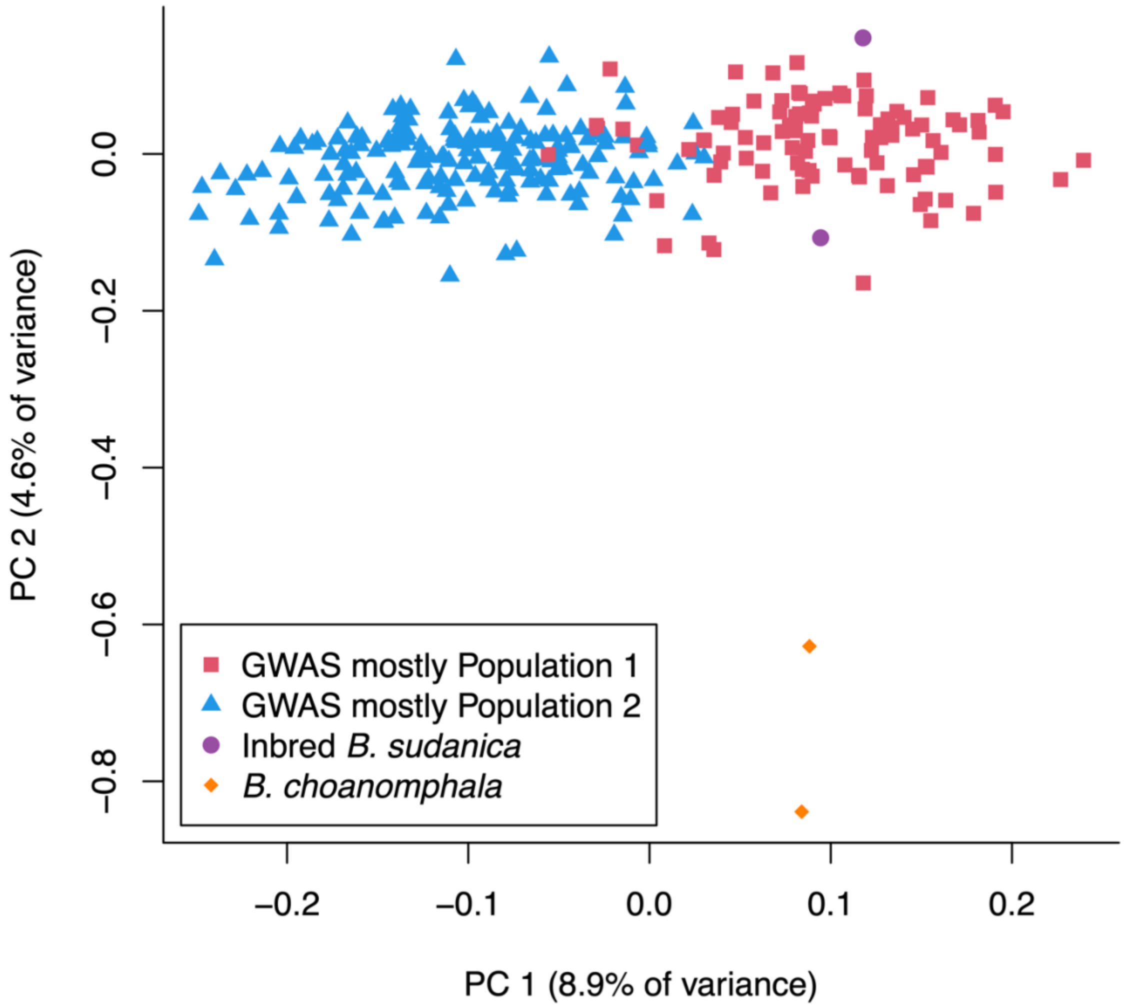

Fig. S7. Principal Component Analysis (n=276) of GWAS *B. sudanica* (color-coded by major ancestry component from ADMIXTURE (15), red squares and blue triangles) along with two outbred *B. choanomphala* (orange diamonds) and two previously sequenced (10) *B. sudanica* inbred lines (purple circles). The first principal component (x-axis) separates the GWAS snails by ancestry, and this is orthogonal to the second principal component (y-axis) that separates *B. sudanica* from *B. choanomphala*. Thus, the population structure in the GWAS snails is not reflective of this interspecies difference.

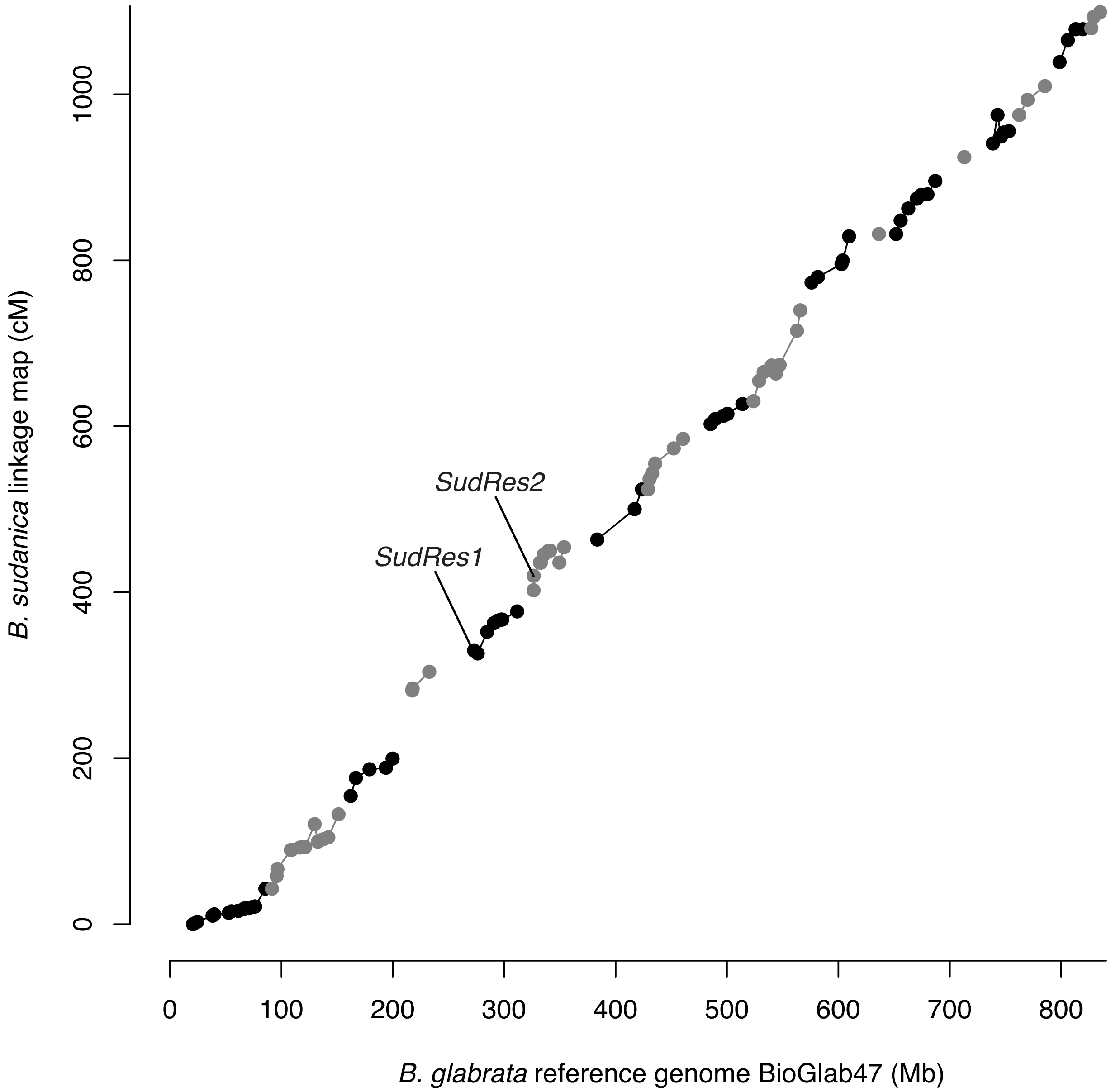

Fig. S8. *Biomphalaria sudanica* linkage map generated from 87 *B. sudanica* F2s generated from two parents from *B. sudanica* inbred lines 163 and KEMRI (20), aligned to *B. glabrata* reference genome xgBioGlab47.1 (Accession GCF_947242115.1). Chromosomes/linkage groups are alternately colored black and gray. The locations of the two validated GWAS loci associated with schistosome resistance in *B. sudanica* in the current study, *SudRes1* and *SudRes2*, are indicated.

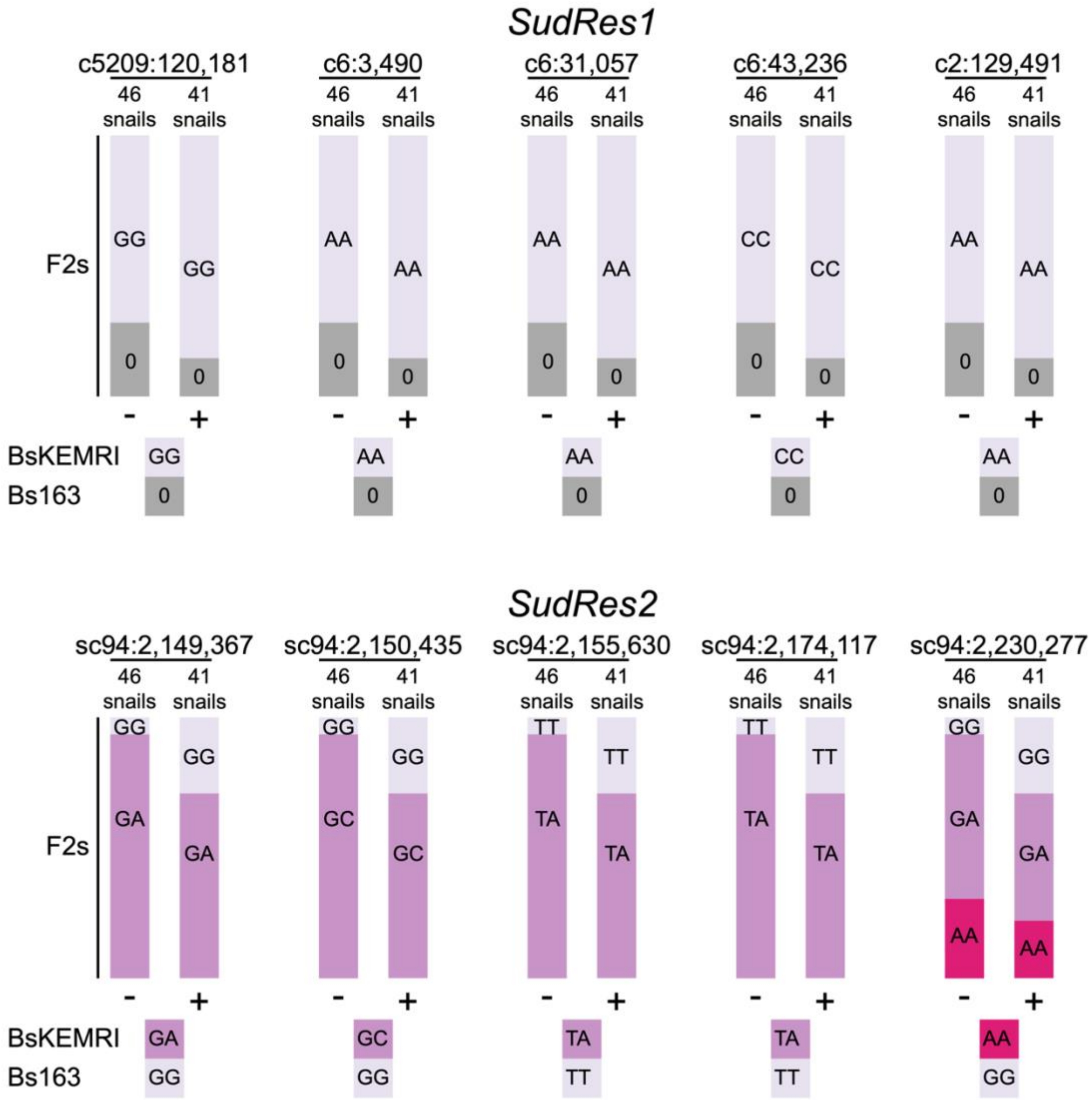

Fig. S9. Associations between validated GWAS loci and resistance phenotype in the linkage cross. For markers across *SudRes1* (top), we did not observe Mendelian segregation but rather a null haplotype resulting in missing data for some offspring and the Bs163 parent, consistent with a large deletion (represented by 0). There was perfect linkage disequilibrium across markers for these missing genotypes, so they do not appear to be genotyping artifacts. This deletion was not significantly correlated with infection, possibly because the resistance allele did not occur in this cross, although the odds of infection was (non-significantly) more than two-fold higher for snails carrying the non-deleted BsKEMRI haplotype. At *SudRes2* (bottom), there was also a haplotype with perfect consistency across markers showing non-Mendelian segregation (except at sc94:2,230,277). This *SudRes2* haplotype was not a null allele, but rather it was never observed as homozygous, indicating it may be a duplication. It was significantly correlated with infection (Fisher's exact test, n=87 F2 offspring, p = 0.009), in the same direction as observed in the GWAS and validation snails (i.e. allele A at sc94:2,174,117 is protective, Fig. 2D). Odds of infection for progeny snails carrying the BsKEMRI haplotype was 5.9 times lower than for progeny snails without it.

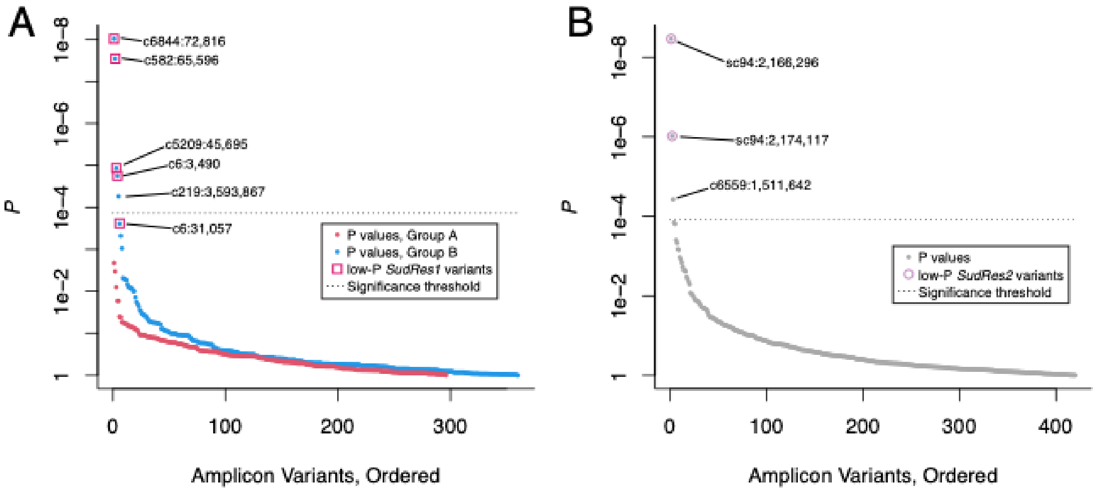

Fig. S10. Validation analyses accounting for kinship with linear mixed models (LMM), as an alternative to the main validation analyses (Fig. 2). (A) LMM *p* values per variant of genotyped-validation samples within ancestry groups, incorporating a kinship matrix. Several *SudRes1* variants are significant validated-variants (uncorrected two-sided *p* = 1e-08, 3e-08, 1e-05, and 2e-05; significance threshold shown by dotted line) within ancestry Group B (blue dots, n=34 biological replicates), as seen in our main analysis that ignores kinship (Fig. 2B). A marker unlinked to *SudRes1* or *SudRes2*, c219:3,593,867, is also significant (uncorrected two-sided *p* = 6e-05), and this contig was among the top outliers in pooled-GWAS (Fig. 1); however we have not pursued this marker further as the signal was not sufficiently robust to appear in our main analysis. (B) LMM *p* values per variant of genotyped-validation samples, accounting for both ancestry and kinship (n=220 biological replicates). *SudRes2* variants sc94:2,166,296 and sc94:2,174,117 are significant validated-variants (uncorrected two-sided *p* = 3e-09 and 1e-06; significance threshold shown by dotted line), consistent with our main analysis (Fig. 2C). A marker unlinked to *SudRes1* or *SudRes2*, c6559:1,511,642, is also significant (uncorrected two-sided *p* = 4e-05); however we have not pursued this marker further as the signal was not sufficiently robust to appear in our main analysis.

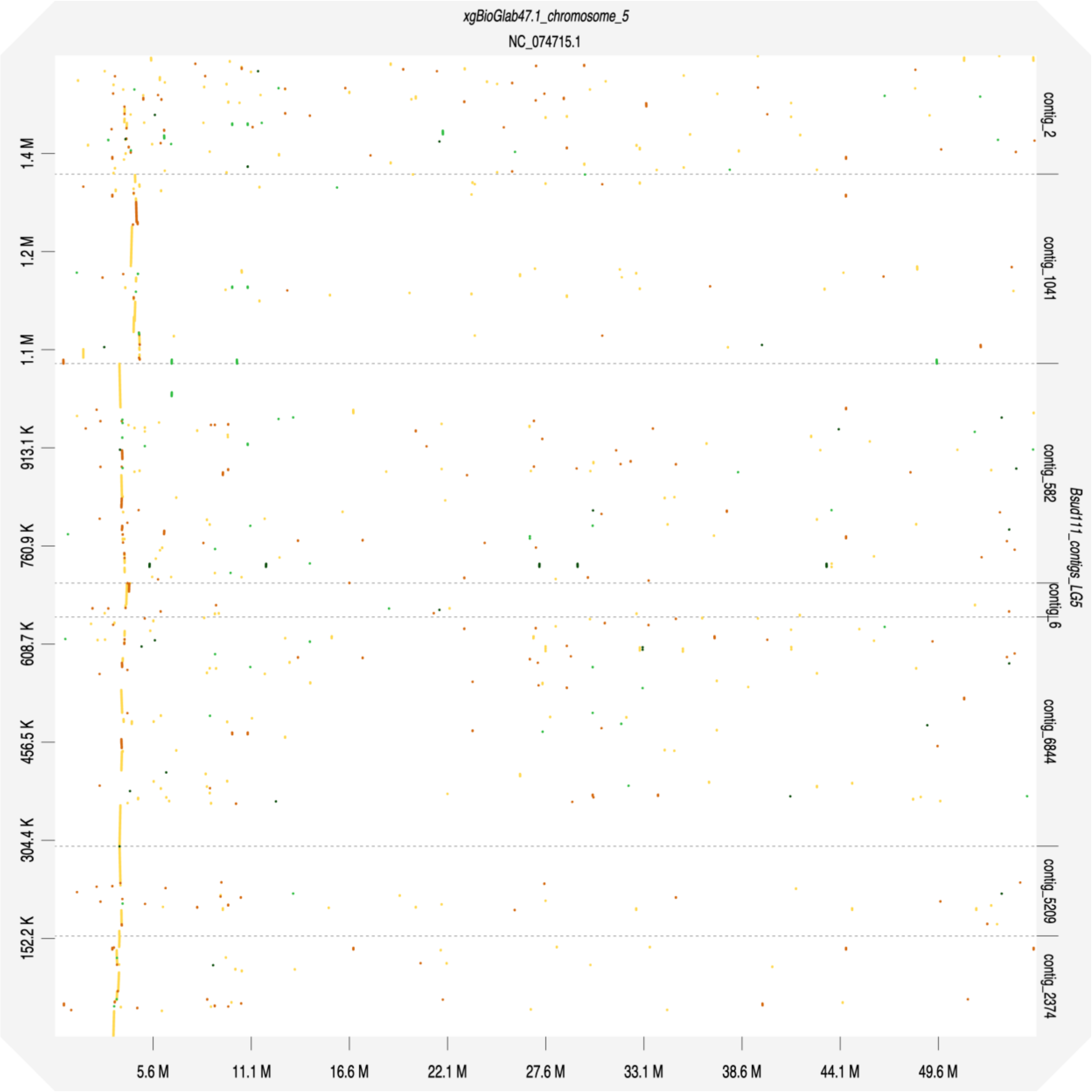

Fig. S11. Dot plot constructed using D-GENIES (23) comparing *Biomphalaria sudanica* genome (10) *SudRes1* region and its neighboring contigs against the *B. glabrata* genome (xgBioGlab47.1, Accession GCF_947242115.1) complete chromosome 5 sequence. Line color represents D-GENIES identity: yellow = 0 to <0.25; orange = 0.25 to <0.50; light green = 0.5 to <0.75; dark green = 0.75 to 1.

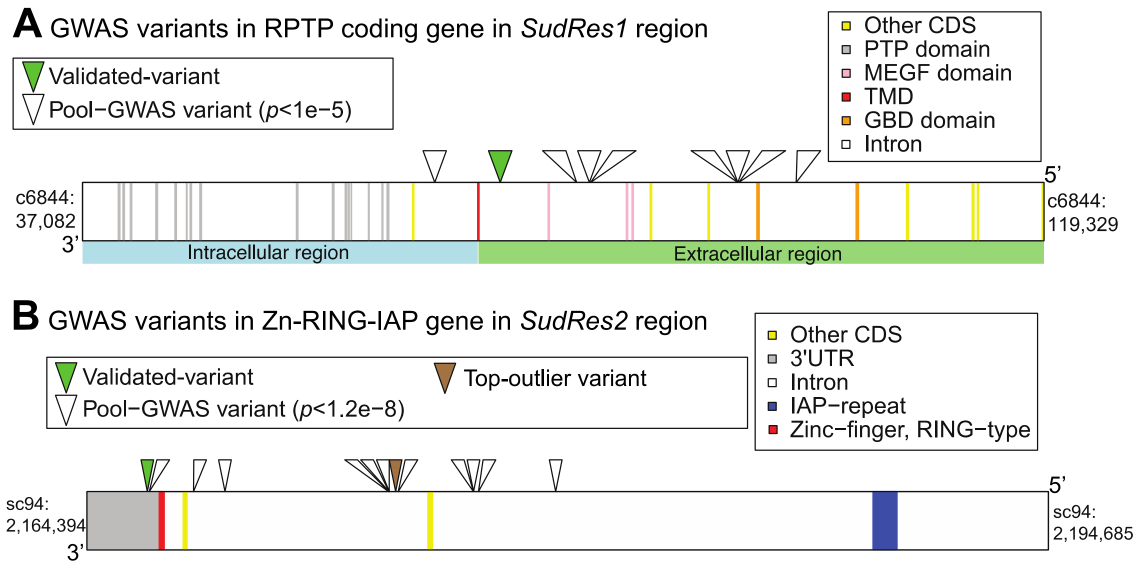

Fig S12. Position of validated-variants in *SudRes1* and *SudRes2* relative to full gene sequences, including introns, coding sequence (CDS) and CDS functional domains. (A) Plot showing nucleotide positions of CDS and CDS functional domains of a receptor-like tyrosine-specific protein phosphatase (RPTP) coding gene (BSUD.17727, Dataset S5) present in the *SudRes1* region of the *B. sudanica* genome. CDS of single pass transmembrane domain (TMD), multiple epidermal growth factor (MEGF) and galactose-binding like (GBD) domains are shown. Also shown are the nucleotide position of the validated-variant in c6844 (see Fig. 2B) and surrounding pooled-GWAS variants with p < 1e-5 (pooled-GWAS Fisher's exact tests), all in introns. (B) Plot showing nucleotide positions of CDS and CDS functional domains within Zinc-finger-RING-type, inhibitor of apoptosis repeat (Zn-RING-IAP) containing gene (BSUD.25704, Dataset S7). Also shown are the nucleotide position of the validated-variant and top-outlier variant (see additive regression model Fig. 2C) and surrounding pooled-GWAS variants with p < 1.2e-8 (pooled-GWAS Fisher's exact tests; 28 other variants with p < 1e-5 not shown for display purposes), all in introns and 3'UTR regions.

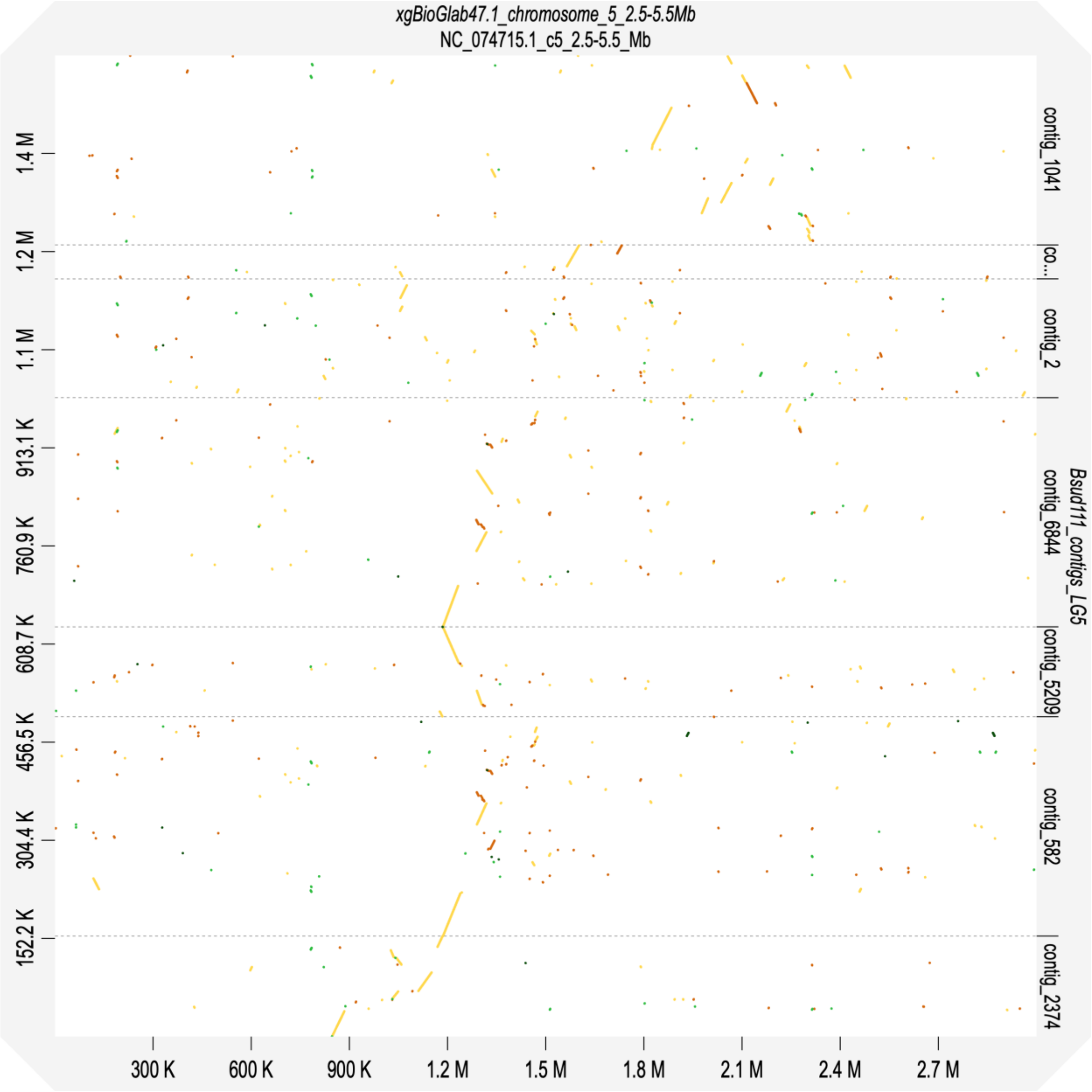

Fig. S13. Dot plot constructed using D-GENIES (23) comparing synteny of *Biomphalaria sudanica* Bs111 genome (10) *SudRes1* region (contigs c6884, c582, c5209, c6, c2) and neighboring contigs (c1041 and c2374) against the *Biomphalaria glabrata* genome (xgBioGlab47.1, Accession GCF_947242115.1) 2.5 – 5.5 Mb region of chromosome 5. Line color represents D-GENIES identity: yellow = 0 to <0.25; orange = 0.25 to <0.50; light green = 0.5 to <0.75; dark green = 0.75 to 1.

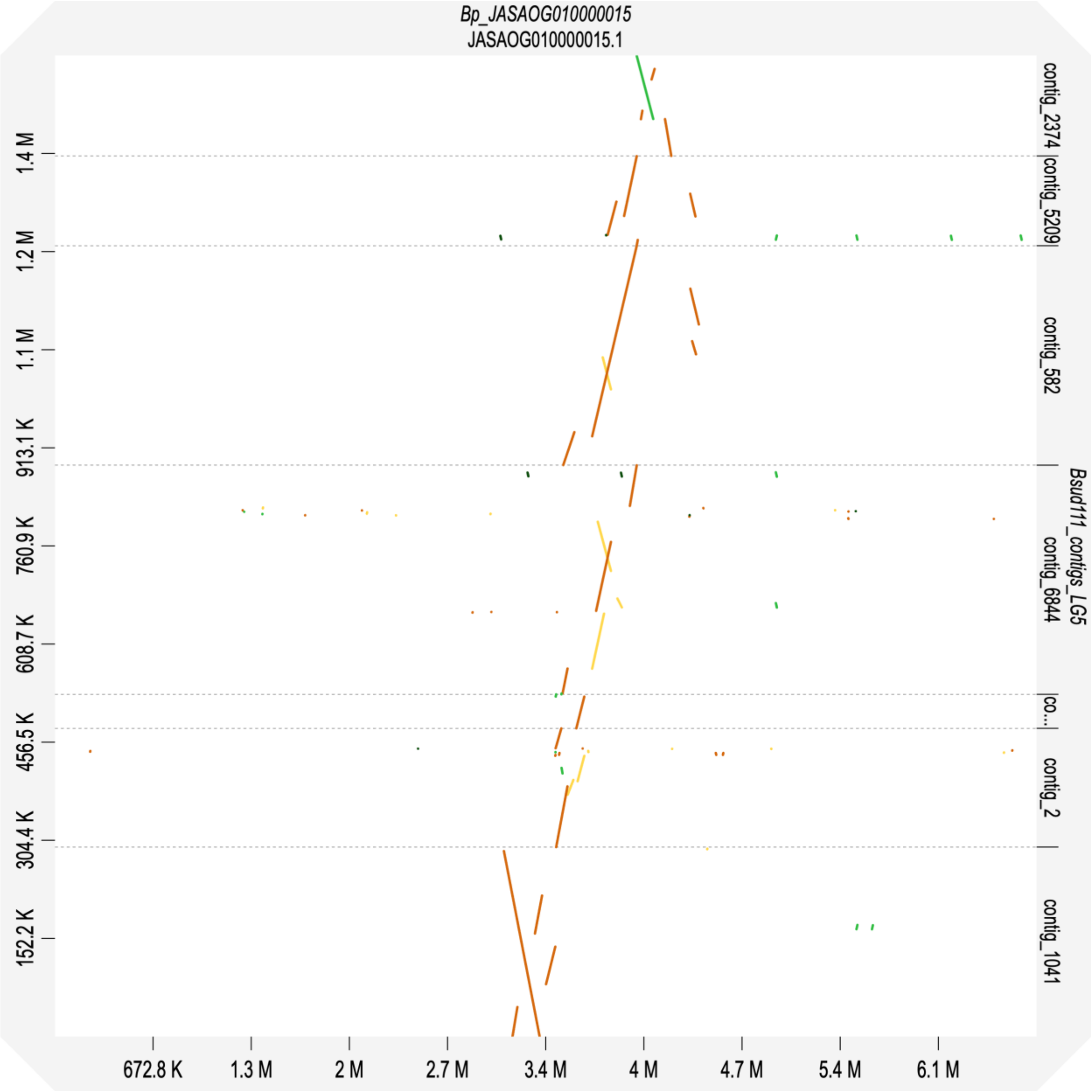

Fig. S14. Dot plot constructed using D-GENIES (23) showing synteny of *Biomphalaria sudanica* Bs111 genome (10) *SudRes1* region (contigs c6884, c582, c5209, c6, c2) and neighboring contigs (c1041 and c2374) against the *Biomphalaria pfeifferi* genome (UNM_Bpfe_1.0, GenBank GCA_030265305.1) orthologous contig JASAOG010000015. Line color represents D-GENIES identity: yellow = 0 to <0.25; orange = 0.25 to <0.50; light green = 0.5 to <0.75; dark green = 0.75 to 1.

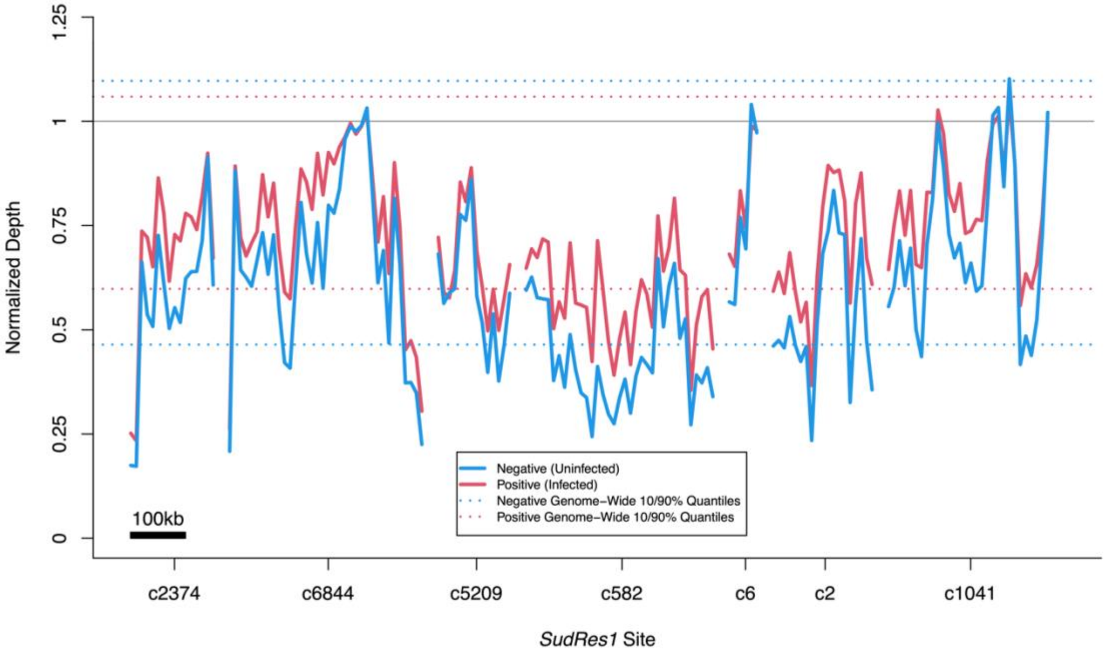

Fig. S15. Aligned read coverage, shown as normalized mean read depth, in 10 kb windows of pooled-GWAS data for the negative (blue) and positive (red) pools (both technical replicates combined) in the *SudRes1* genomic region (c6844, c5209, c582, c6, c2) and neighboring contigs. Depths are scaled to the genome-wide median for each pool (= 1, solid grey line). Across all *SudRes1* contigs, coverage is relatively low, often less than the 10% quantile for the genome (dotted lines).

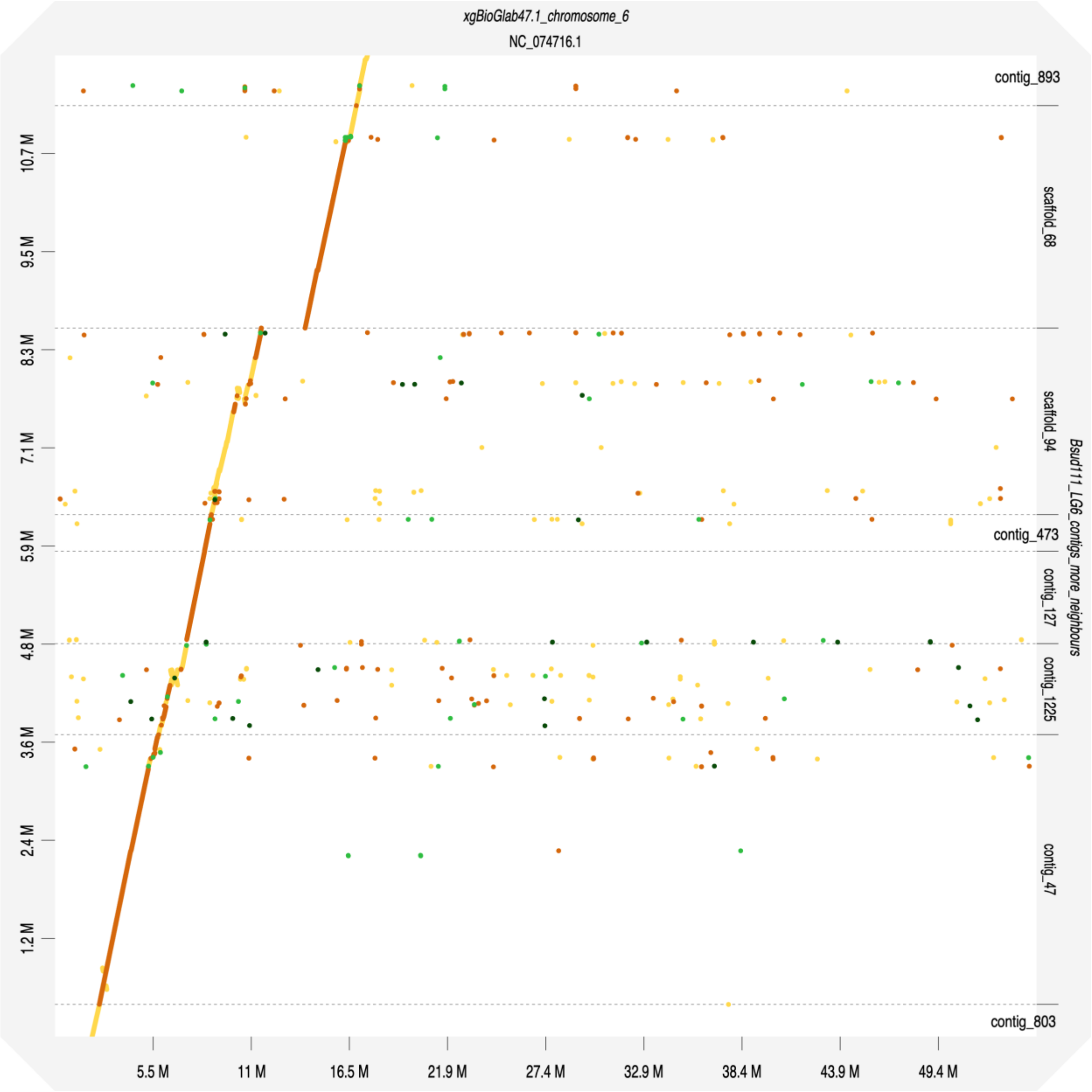

Fig. S16. Dot plot constructed using D-GENIES (23) comparing *Biomphalaria sudanica* genome (10) *SudRes2* region (contig sc94: 1.82 – 2.26 Mb) and its neighboring regions/contigs against the *Biomphalaria glabrata* genome xgBioGlab47.1 (Accession GCF_947242115.1) complete chromosome 6 sequence. Line color represents D-GENIES identity: yellow = 0 to <0.25; orange = 0.25 to <0.50; light green = 0.5 to <0.75; dark green = 0.75 to 1.

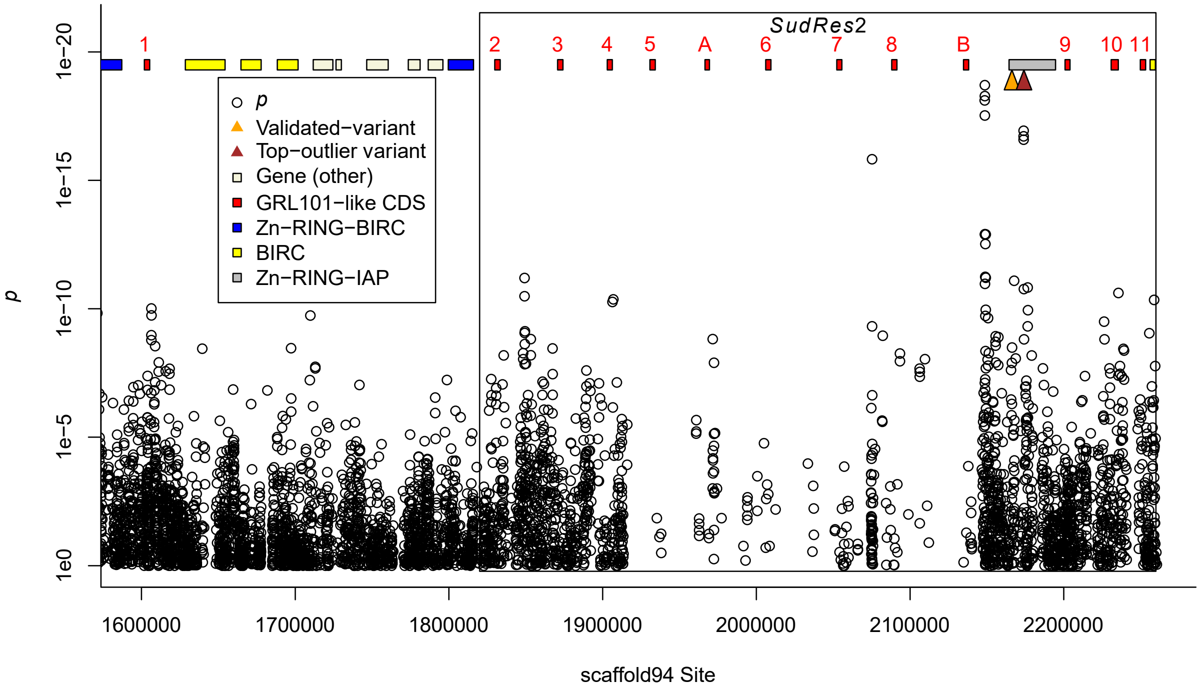

Fig. S17. Enlargement of contig Bs111 sc94 (1.6 Mb to end, displayed in forward direction) on *Biomphalaria sudanica* chromosome 6, showing Fisher's exact test *p* values for all pooled-GWAS variants. The *SudRes2* region (represented by the black box surrounding sc94:1.82-2.26 Mb), contains 23 pooled-GWAS dual-variants with Fisher's test $p \leq$ 5e-11, as well as the validated-variant (sc94:2,166,296, see Fig. 2C and 2D) and top-outlier variant (sc94:2,174,117, see Fig. 2C and 2D) associated with *B. sudanica* resistance to *Schistosoma mansoni*. Positions of the 13 (12 in *SudRes2*) GRL101-like G protein coupled receptor coding sequences (GRL101-like CDS) in sc94 are shown, as well as positions of genes containing combinations of zinc finger RING-type (Zn-RING), inhibitor of apoptosis (IAP) and baculoviral IAP repeat containing (BIRC) domains.

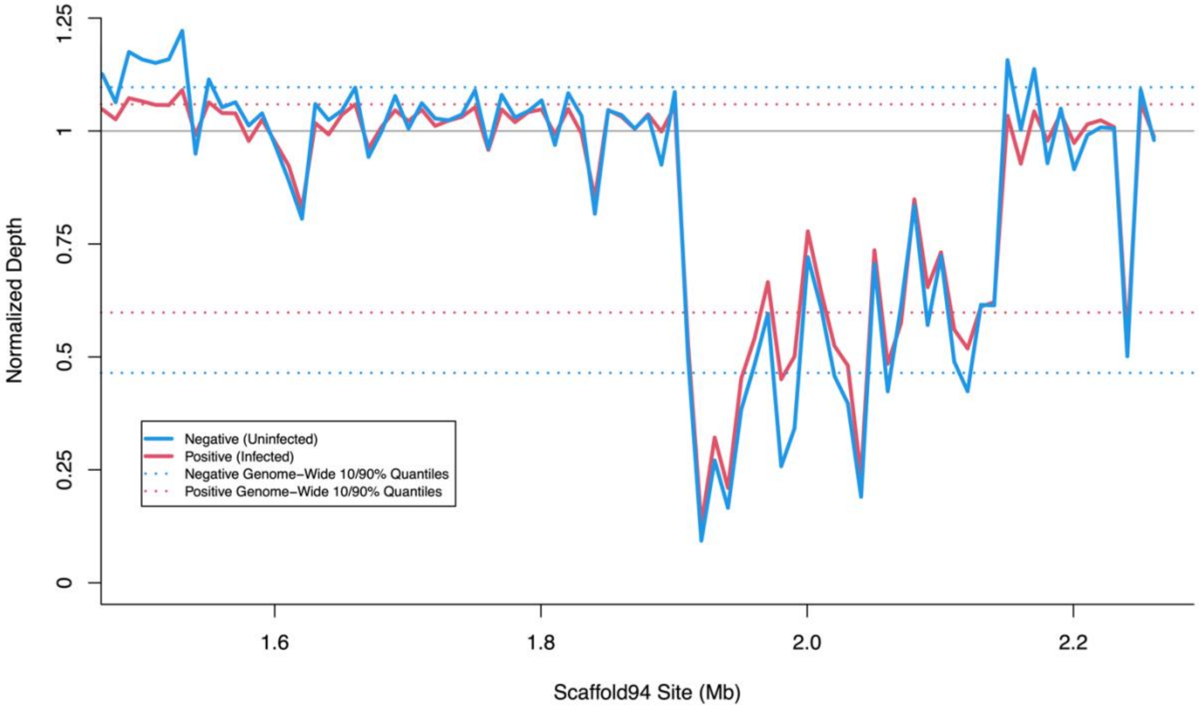

Fig. S18. Aligned read coverage, shown as normalized mean read depth, in 10 kb windows of pooled-GWAS data for the negative and positive pools (both technical replicates combined). Depths are scaled to the genome-wide median for each pool (= 1, solid grey line). The *SudRes2* gene region where a cluster of GRL101-like GPCR proteins were manually annotated lies between 1.6 Mb to the end of the contig sc94, corresponding to the region where read depth drops.

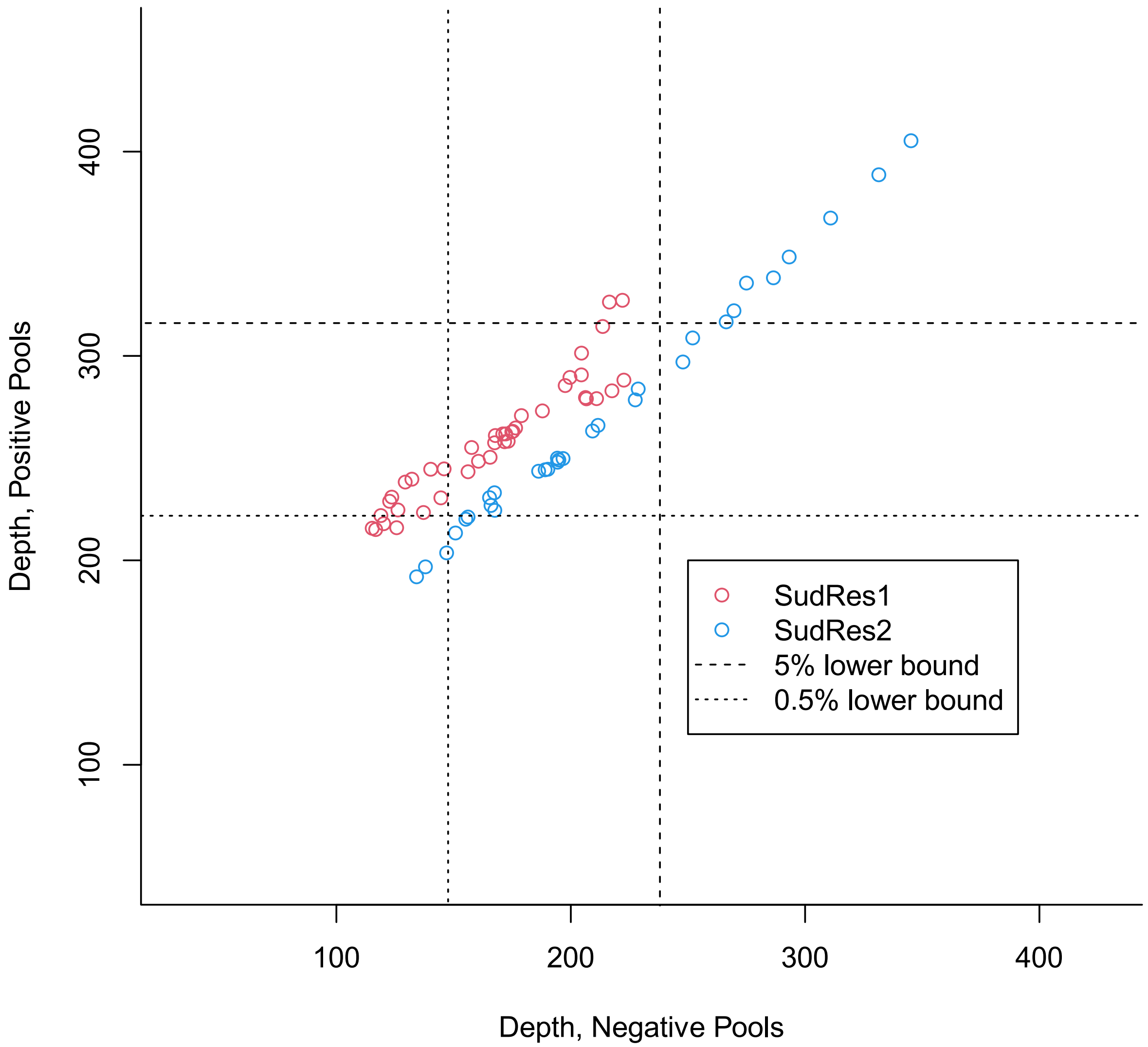

Fig. S19. Genome-wide pooled-GWAS mean depths in sliding 100 kb windows (step size = 10 kb) for the negative and positive pools (both technical replicates combined). Both *SudRes1* and *SudRes2* are notable for showing unusually low depth as well as slightly skewed ratios of positive/negative depth (unusually high for *SudRes1* and unusually low for *SudRes2*). Low coverage may indicate these regions are particularly variable and dynamic, with large sequence difference from the reference genome and/or poor mappability among highly similar adjacent paralogs.

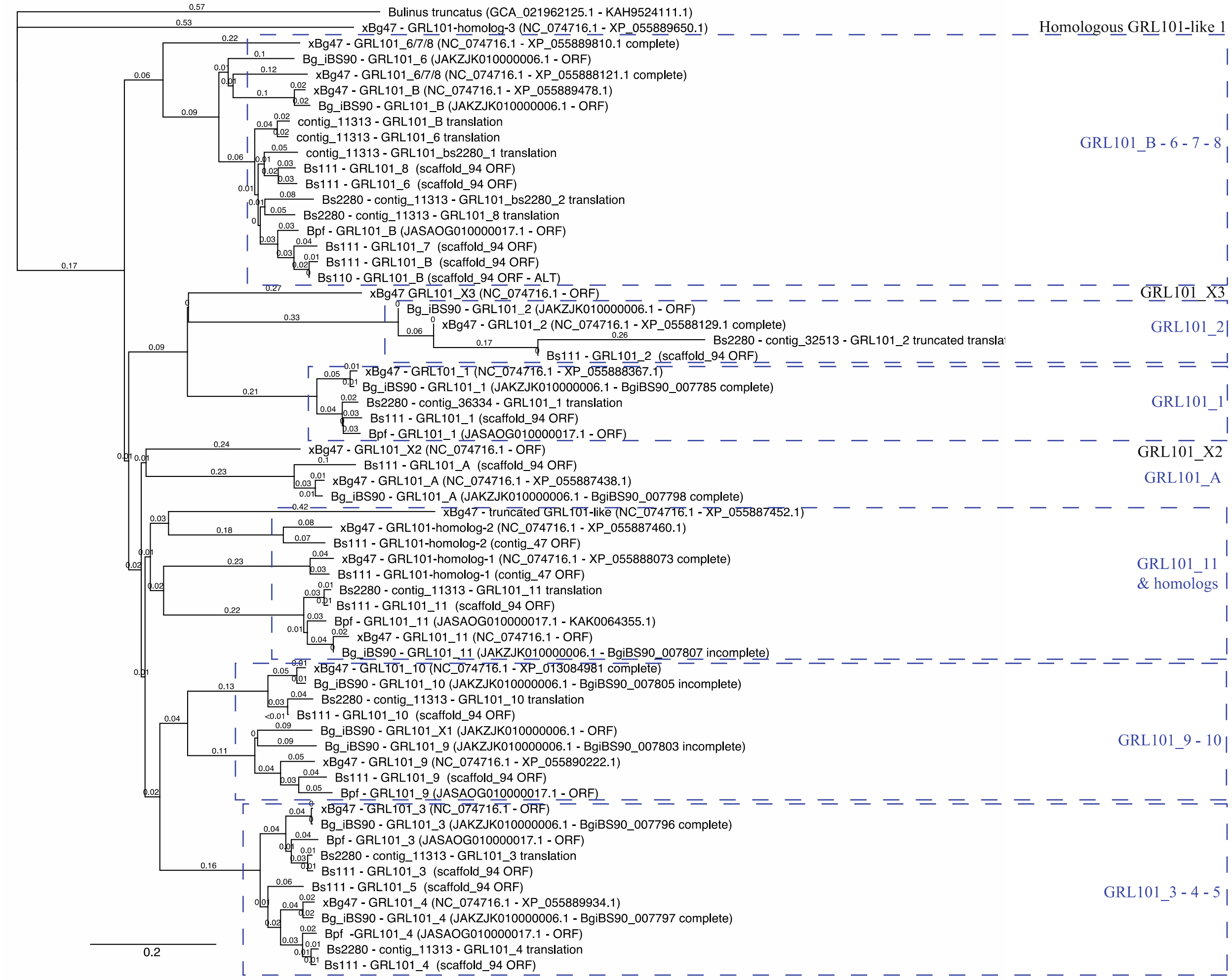

Fig. S20. Neighbor-Joining tree (model Jukes-Cantor) of all GRL101-like G protein coupled receptor (GPCR) protein sequences identified in *B. sudanica* (Bs111 and Bs2280), *B. pfeifferi* (Bpf (29)) *B. glabrata* iBs90 (Bg_iBs90 (13)) and *B. glabrata* BG47 (xBioGlab47, NCBI RefSeq: GCF_947242115.1) *SudRes2* and adjacent regions. Amino acid sequences aligned using the Geneious Alignment tool and tree constructed using the Geneious tree builder. Branch length shown reflects the number of substitutions per site. The outgroup is a GRL101-GPCR orthologous protein in *Bulinus truncatus* (GenBank Accessions: KAH9524111.1 (30)). Gene xBglab47-XP_055887452.1 is truncated (no transmembrane domain region). GRL101_1 and the homologous GRL101-like genes represent those not contained within the *SudRes2* region of *B. sudanica* (sc94:1.82-2.26 Mb) or *B. glabrata* (GCF_947242115.1, 8.74-9.07 Mb). An additional alternative protein isoform is given for *B. sudanica* protein GRL101_B, that was generated from transcript alignments of inbred line Bs110 (10).

SI References

1. D. H. Alexander, J. Novembre, K. Lange, Fast model-based estimation of ancestry in unrelated individuals. *Genome Res.* **19**, 1655–1664 (2009).
2. M. P. Conomos, M. B. Miller, T. A. Thornton, Robust Inference of Population Structure for Ancestry Prediction and Correction of Stratification in the Presence of Relatedness. *Genet. Epidemiol.* **39**, 276–293 (2015).
3. T. Pennance, *et al.*, The genome and transcriptome of the snail *Biomphalaria sudanica s.l.*: Immune gene diversification and highly polymorphic genomic regions in an important African vector of *Schistosoma mansoni*. *BMC Genomics* **25**, 192 (2024).
4. J. M. Spaan, *et al.*, Multi-strain compatibility polymorphism between a parasite and its snail host, a neglected vector of schistosomiasis in Africa. *Curr. Res. Parasitol. Vector-Borne Dis.* **3**, 100120 (2023).
5. F. Cabanettes, C. Klopp, D-GENIES: dot plot large genomes in an interactive, efficient and simple way. *PeerJ* **6**, e4958 (2018).
6. L. Bu, *et al.*, A genome sequence for *Biomphalaria pfeifferi*, the major vector snail for the human-infecting parasite *Schistosoma mansoni*. *PLoS Negl. Trop. Dis.* **17**, e0011208 (2023).
7. L. Bu, *et al.*, Compatibility between snails and schistosomes: insights from new genetic resources, comparative genomics, and genetic mapping. *Commun. Biol.* **5**, 940 (2022).
8. N. D. Young, *et al.*, Nuclear genome of *Bulinus truncatus*, an intermediate host of the carcinogenic human blood fluke *Schistosoma haematobium*. *Nat. Commun.* **13**, 977 (2022).
9. G. R. A. Margarido, A. P. Souza, A. A. F. Garcia, OneMap: software for genetic mapping in outcrossing species. *Hereditas* **144**, 78–79 (2007).
