# Supplementary material for: Genes linked to schistosome resistance identified in a genome-wide association study of African snail vectors
Source: Nat Commun. 2025 Jul 27;16:6918. doi: 10.1038/s41467-025-61760-8 (PMC12297450; doi:10.1038/s41467-025-61760-8)
Supplement: Supplementary file 2 — Description Of Additional Supplementary File [file 41467_2025_61760_MOESM2_ESM.pdf]

Description of Additional supplementary files

Supplementary Data 1

Raw and processed sequence data statistics, showing number of Illumina PE150 reads obtained from pooled-GWAS sequencing of positive and negative snails, including technical replicates, and the final number of reads retained following bioinformatic processing that were aligned to the Biomphalaria sudanica genome.

Supplementary Data 2

Resulting data from Biomphalaria sudanica linkage map analysis using 210 markers analyzed with OneMap v. 3.0.0 (1), and showing relative chromosomes and positions on the Biomphalaria glabrata genome xgBioGlab47.1 (Accession GCF_947242115.1).

Supplementary Data 3

Markers used in the Amplicon panel for validating pooled-GWAS variants, a priori gene candidates and linkage markers. Dataset includes target variant coordinates (Contig/Site) in the B. sudanica genome (2), the purpose of the marker inclusion (dualvariant, singleton-variant, a priori gene candidate, linkage map marker) and the forward and reverse primer sequences. For each marker (except for all linkage map markers that are not applicable for this analysis) results of a dominance model (Fisher's exact tests) are given for genotyped-validation snails divided between groups A and B representing snail ancestry (majority population 1 and 2, respectively), and the results of an Additive Regression model including all genotyped-validation snails.

Supplementary Data 4

Genotype data per locus and per marker for amplicon panel B. sudanica. Rows are amplicon loci and columns are individual snails. Snails are categorized as “GWAS” (genotyped-pooled-GWAS snails used in pooled sequencing) or “validation” (genotyped-validation snails independent from pooled sequencing), and with infection status of “true positive”, “true negative”, or “false negative”. Ancestry is shown as the proportion originating from Population 2. Close relatives (kinship ≥ 0.1) of each snail are indicated. In the genotype matrix, alleles are arbitrarily designated as “first” or “second”, and numbers indicate the count of second alleles (i.e. 0 = first allele homozygous, 1 = heterozygous, 2 = second allele homozygous). NA indicates missing genotypes. The Sman_16S marker is counted as “Present” if at least 50 reads were detected from this locus, though we did not use this information for phenotyping.

Supplementary Data 5

Gene identifiers and associated information for Biomphalaria sudanica (Bs111 and Bs2280) genes in SudRes1 region, including orthologous gene identifiers from the B. glabrata iBS90 genome (3). NA represents missing information where information for specific genes (i.e. Biomphalaria spp. gene orthologs or gene coordinates) were not identified in the current study.

Supplementary Data 6

Genotypes identified at amplicon panel variants in SudRes1 and SudRes2 regions of susceptible and resistant Biomphalaria sudanica snails. Contig and SNP locations are provided for the B. sudanica reference genome (Bs111) and the resistant GWAS snail (Bs2280), including the location of SNPs targeted at alternative contigs also amplified by respective amplicon panel primers.

Supplementary Data 7

Gene identifiers and associated information for Biomphalaria sudanica (Bs111 and Bs2280) genes in SudRes2 region, including orthologous gene identifiers, or orthologous gene coordinates, of GRL101 genes identified in B. glabrata iBS90 (3), B. glabrata xgBioGlab47.1 (Accession GCF_947242115.1) and B. pfeifferi (4) genome assemblies. NA represents missing information where information for specific genes (i.e. Biomphalaria spp. gene orthologs or gene coordinates) were not identified in the current study.
