# Supplementary material for: Genes linked to schistosome resistance identified in a genome-wide association study of African snail vectors
Source: Nat Commun. 2025 Jul 27;16:6918. doi: 10.1038/s41467-025-61760-8 (PMC12297450; doi:10.1038/s41467-025-61760-8)
Supplement: Supplementary file 10 — Reporting summary [file 41467_2025_61760_MOESM10_ESM.pdf]

Corresponding author(s): Michelle Steinauer

Last updated by author(s): 2025/5/22

Reporting Summary

Nature Portfolio wishes to improve the reproducibility of the work that we publish. This form provides structure for consistency and transparency in reporting. For further information on Nature Portfolio policies, see our Editorial Policies and the Editorial Policy Checklist.

Statistics

For all statistical analyses, confirm that the following items are present in the figure legend, table legend, main text, or Methods section.

n/a	Confirmed	
☐	☒	The exact sample size (*n*) for each experimental group/condition, given as a discrete number and unit of measurement
☐	☒	A statement on whether measurements were taken from distinct samples or whether the same sample was measured repeatedly
☐	☒	The statistical test(s) used AND whether they are one- or two-sided<br>*Only common tests should be described solely by name; describe more complex techniques in the Methods section.*
☐	☒	A description of all covariates tested
☐	☒	A description of any assumptions or corrections, such as tests of normality and adjustment for multiple comparisons
☐	☒	A full description of the statistical parameters including central tendency (e.g. means) or other basic estimates (e.g. regression coefficient) AND variation (e.g. standard deviation) or associated estimates of uncertainty (e.g. confidence intervals)
☐	☒	For null hypothesis testing, the test statistic (e.g. *F*, *t*, *r*) with confidence intervals, effect sizes, degrees of freedom and *P* value noted<br>*Give P values as exact values whenever suitable.*
☒	☐	For Bayesian analysis, information on the choice of priors and Markov chain Monte Carlo settings
☒	☐	For hierarchical and complex designs, identification of the appropriate level for tests and full reporting of outcomes
☐	☒	Estimates of effect sizes (e.g. Cohen's *d*, Pearson's *r*), indicating how they were calculated

Our web collection on statistics for biologists contains articles on many of the points above.

Software and code

Policy information about availability of computer code

Data collection	n/a

Data analysis	All custom perl scripts used in the bioinformatic analysis are available in the project repository: www.figshare.com/projects/Genome_Wide_Association_Study_of_Biomphalaria_sudanica/230615.<br>The following published software was used:<br>cutadapt v3.1 (Martin 2011)<br>trimmomatic v0.30 (Bolder et al. 2014)<br>bwa v0.7.17 (Li and Durbin 2009)<br>samtools v1.19.2 (Li et al. 2009)<br>bcftools v1.9 (Li et al. 2011)<br>ADMIXTURE 1.3.0 (Alexander et al. 2009)<br>BLAST v2.14.0+ and v2.15.0+ (Altschul et al. 1990)<br>SNPRelate v1.40.0 (Zheng et al. 2012)<br>PC-AiR in GENESIS v2.36.0 (Conomos et al. 2015)<br>gaston v1.6 (Perdry et al. 2023)<br>OneMap v3.0.0 (Margarido et al. 2007)<br>R v4.3 (R Core Team 2018)<br>Flye v2.9.4 (Kolmogorov et al. 2019)<br>Geneious v2022.0.2 (Biomatters Ltd.)

For manuscripts utilizing custom algorithms or software that are central to the research but not yet described in published literature, software must be made available to editors and reviewers. We strongly encourage code deposition in a community repository (e.g. GitHub). See the Nature Portfolio guidelines for submitting code & software for further information.

Data

Policy information about availability of data

All manuscripts must include a data availability statement. This statement should provide the following information, where applicable:

- Accession codes, unique identifiers, or web links for publicly available datasets
- A description of any restrictions on data availability
- For clinical datasets or third party data, please ensure that the statement adheres to our policy

All sequence data, including PacBio HiFi raw reads from genome sequence data of B. sudanica Bs2280, has been uploaded onto the NCBI SRA under BioProject PRJNA1149315 with BioSample accessions SAMN43241892, SAMN43241893, SAMN43241894, SAMN43241895, SAMN45084274. The genome assembly of Bs2280. We also include six supplementary datasets along with submission.

Research involving human participants, their data, or biological material

Policy information about studies with human participants or human data. See also policy information about sex, gender (identity/presentation), and sexual orientation and race, ethnicity and racism.

Reporting on sex and gender	Subjects of both sexes were recruited into the study. Since we did not collect data on the human participants, and only used the eggs from their schistosome burdens, the enrollment of subjects according to sex or gender will not effect the study results as we are reporting on the snail vector hosts.
Reporting on race, ethnicity, or other socially relevant groupings	We enrolled individuals attending primary schools near the shores of Lake Victoria in schistosomiasis transmission zones. The population here is predominantly Kenyan of the Luo tribe. Humans were enrolled solely to collect the eggs of schistosomes circulating in this endemic area, we solely collected data regarding infection status so that we could followup with infected individuals to provide treatment with praziquantel.
Population characteristics	We enrolled individuals attending primary schools near the shores of Lake Victoria in schistosomiasis transmission zones between the ages of 6-15 years, as this is the population that is most heavily infected with schistosomiasis.
Recruitment	Inclusion criteria include children 6-15 years old, participants of both sexes, resident in the study area for at least 1 year prior to commencement of the study and the willingness by parents/LAR to provide written informed consent and willingness of children to participate in the study (to provide assent). Exclusion criteria include persons who have received praziquantel less than 2 months prior to sampling, persons who are observably ill or has an underlying/abnormal medical condition, and a child unwilling to provide assent, or child whose parent does not consent to the study. To recruit individuals and obtain parental consent, we worked with school officials. Because participation is voluntary, there may be some bias in our representation of humans that are infected; however, we expect that each participant has recruited a random sample of schistosomes. Therefore, we do not expect that any selection bias of human participation would have a strong impact on the results of our study which aimed to ascertain the genetics of vector resistance to the parasites.
Ethics oversight	Collections of S. mansoni from schoolchildren were approved by KEMRI's Scientific and Ethics Review Unit (SERU), reference SERU No. 3540, by Institutional Review Board of the Western University of Health Sciences No. FB19/IRB/111 2021, the and by the Institutional Review Board of the University of New Mexico (UNM) No.18115.

Note that full information on the approval of the study protocol must also be provided in the manuscript.

Field-specific reporting

Please select the one below that is the best fit for your research. If you are not sure, read the appropriate sections before making your selection.

☐ Life sciences ☐ Behavioural & social sciences ☒ Ecological, evolutionary & environmental sciences

For a reference copy of the document with all sections, see nature.com/documents/nr-reporting-summary-flat.pdf

Ecological, evolutionary & environmental sciences study design

All studies must disclose on these points even when the disclosure is negative.

Study description	We performed a genome wide association study on the snail vector of schistosomiasis, Biomphalaria sudanica, to determine the regions of the genome associated with the phenotype of resistance or susceptibility. We collected snails from a wild population and bred them in the laboratory. Snails were exposed to Schistosoma mansoni (dose = 8) and then determined to be infected or not by microscopy and PCR. The initial GWAS was a pooled design in which we pooled DNA from all infected (susceptible) snails and all unininfected snails (resistant) and compared SNP allele frequencies between the pools. To validate any outlier SNPs, we designed an amplicon panel to genotype individuals at outlier SNPs. We corrected for family structure within the analysis to be sure potential relatedness was not introducing bias.
Research sample	We collected snails, Biomphalaria sudanica, from a hotspot transmission area in Lake Victoria Kenya. The rationale for choosing this species is that it is the primary vector of schistosomiasis in the Lake Victoria Basin, and the sample is meant to reflect the genetics of the wild population. We started with 321 individuals collected from the field and bred them in the laboratory. These snails are hermaphroditic, so each individual is both male and female. The age range is unknown, but comprised adult snails (> 5 mm). Target sample size was chosen following Tennessen et al. 2020 (PMID: 32845238). Our GWAS exposed 1400 F1 snails to the schistosomes. A total of 254 snails died during the prepatent period. Our final design resulted in 615 positive (susceptible) snails, and 393 negative snails (resistant).
Sampling strategy	We sampled snails from Anyanga Beach area in Kanyibok, Lake Victoria, Kenya (Latitude: -00.08958°, Longitude: 34.08592°) using shallow snail scoops. arget sample size was chosen following Tennessen et al. 2020 (PMID: 32845238) though limited by practical logistics.
Data collection	Snails were determined to be positive or negative first by isolating the snails and using microscopy to check for infection. The snails that were deemed positive were confirmed to be so by examining a tissue squash prior to DNA extraction. Negative snails were confirmed to be negative by a PCR that was developed by the team. Data were initially recorded with pen and paper and then transcribed to an excel spreadsheet. These methods were performed by T.P., J.S., M.L.S., G.O., M.M., K.A., M.O.
Timing and spatial scale	A single sample was taken to avoid introducing artifacts due to population structure.
Data exclusions	Snails that had an active trematode infection upon collection were not included in the breeding of F1 snails. Snails that did not survive to a patent infection were excluded. Snails for which adequate high qualtiy DNA could not be obtained were excluded from the sample.
Reproducibility	We performed a validation of the original GWAS using an amplicon panel design.
Randomization	Snails were allocated into groups based on their susceptibility phenotypes, which were determined by whether or not they were observed to shed parasites, and whether or not they tested positive for parasite DNA with a PCR assay. Randomization is not appropriate for this sort of study.
Blinding	Blinding was not possible for pooled-GWAS, since snails sharing phenotypes had to be combined. Validation snails were genotyped by a commercial service (GTseek) with no knowledge of their phenotypes.

Did the study involve field work? ☒ Yes ☐ No

Field work, collection and transport

Field conditions	One field collection of the snails was performed on March 27, 2018. Snails were collected from Lake Victoria via scoops and transported to the laboratory in a plastic container lined with a damp cloth. The team and snails traveled by vehicle.
Location	Anyanga Beach area in Kanyibok, Lake Victoria, Kenya (Latitude: -00.08958°, Longitude: 34.08592°)
Access & import/export	We obtained all the necessary permissions and permits to perform the collections, export snails from Kenya, and import snails into the U.S. Kenya Medical Research Institute (KEMRI) Scientific Review Unit (Approval # KEMRI/RES/7/3/1 and KEMRI/SERU/CGHR/035/3864), Kenya's National Commission for Science, Technology, and Innovation (License # NACOSTI/P/15/9609/4270 and NACOSTI/P/22/14839), Kenya Wildlife Services (permit # 0004754 and # WRTI-0136–02-22), and National Environment, Management Authority (permit # NEMA/AGR/46/2014 – Registration # 0178 and NEMA/AGR/159/2022 – Registration # 201)
Disturbance	Disturbances were not noted. Snails are collected regularly from this area from our team and others, and no decline in the

Disturbance	population due to sampling has been noted. Snail populations are large in Lake Victoria and it is unlikely that removing ~300 snails would negatively impact the population.

Reporting for specific materials, systems and methods

We require information from authors about some types of materials, experimental systems and methods used in many studies. Here, indicate whether each material, system or method listed is relevant to your study. If you are not sure if a list item applies to your research, read the appropriate section before selecting a response.

Materials & experimental systems

n/a	Involved in the study
☒	☐ Antibodies
☒	☐ Eukaryotic cell lines
☒	☐ Palaeontology and archaeology
☐	☒ Animals and other organisms
☒	☐ Clinical data
☒	☐ Dual use research of concern
☒	☐ Plants

Methods

n/a	Involved in the study
☒	☐ ChIP-seq
☒	☐ Flow cytometry
☒	☐ MRI-based neuroimaging

Animals and other research organisms

Policy information about studies involving animals; ARRIVE guidelines recommended for reporting animal research, and Sex and Gender in Research

Laboratory animals	Snails, Biomphalaria sudanica
Wild animals	Snails, Biomphalaria sudanica
Reporting on sex	hermaphroditic, so both sexes equally.
Field-collected samples	The original population of 321 snails were obtained from Anyanga Beach area in Kanyibok, Lake Victoria, Kenya (Latitude: -00.08958°, Longitude: 34.08592°)
Ethics oversight	We obtained all the necessary permissions and permits to perform the collections, export snails from Kenya, and import snails into the U.S. Kenya Medical Research Institute (KEMRI) Scientific Review Unit (Approval # KEMRI/RES/7/3/1 and KEMRI/SERU/CGHR/035/3864), Kenya's National Commission for Science, Technology, and Innovation (License # NACOSTI/P/15/9609/4270 and NACOSTI/P/22/14839), Kenya Wildlife Services (permit # 0004754 and # WRTI-0136–02-22), and National Environment, Management Authority (permit # NEMA/AGR/46/2014 – Registration # 0178 and NEMA/AGR/159/2022 – Registration # 201). The IACUC does not regulate snails.

Note that full information on the approval of the study protocol must also be provided in the manuscript.

Plants

Seed stocks	n/a
Novel plant genotypes	n/a
Authentication	n/a
